# Multicriteria optimization enables less experienced planners to efficiently produce high quality treatment plans in head and neck cancer radiotherapy

**DOI:** 10.1186/s13014-015-0385-9

**Published:** 2015-04-12

**Authors:** Roel GJ Kierkels, Ruurd Visser, Hendrik P Bijl, Johannes A Langendijk, Aart A van ‘t Veld, Roel JHM Steenbakkers, Erik W Korevaar

**Affiliations:** Department of Radiation Oncology, University of Groningen, University Medical Center Groningen, PO Box 30001, Groningen, 9700 RB the Netherlands; Department of Medical Imaging and Radiation Therapy, Hanze University of Applied Sciences, Groningen, the Netherlands

**Keywords:** Multicriteria optimization, Intensity-modulated radiotherapy, Head and neck cancer, Treatment planning

## Abstract

**Objectives:**

To demonstrate that novice dosimetry planners efficiently create clinically acceptable IMRT plans for head and neck cancer (HNC) patients using a commercially available multicriteria optimization (MCO) system.

**Methods:**

Twenty HNC patients were enrolled in this *in-silico* comparative planning study. Per patient, novice planners with less experience in dosimetry planning created an IMRT plan using an MCO system (RayStation). Furthermore, a conventionally planned clinical IMRT plan was available (Pinnacle^3^). All conventional IMRT and MCO-plans were blind-rated by two expert radiation-oncologists in HNC, using a 5-point scale (1–5 with 5 the highest score) assessment form comprising 10 questions. Additionally, plan quality was reported in terms of planning time, dosimetric and normal tissue complication probability (NTCP) comparisons. Inter-rater reliability was derived using the intra-class correlation coefficient (ICC).

**Results:**

In total, the radiation-oncologists rated 800 items on plan quality. The overall plan score indicated no differences between both planning techniques (conventional IMRT: 3.8 ± 1.2 vs. MCO: 3.6 ± 1.1, *p* = 0.29). The inter-rater reliability of all ratings was 0.65 (95% CI: 0.57–0.71), indicating substantial agreement between the radiation-oncologists. In 93% of cases, the scoring difference of the conventional IMRT and MCO-plans was one point or less. Furthermore, MCO-plans led to slightly higher dose uniformity in the therapeutic planning target volume, to a lower integral body dose (13.9 ± 4.5 Gy vs. 12.9 ± 4.0 Gy, *p <* 0.001), and to reduced dose to the contra-lateral parotid gland (28.1 ± 11.8 Gy vs. 23.0 ± 11.2 Gy, *p <* 0.002). Consequently, NTCP estimates for xerostomia reduced by 8.4 ± 7.4% (*p* < 0.003). The hands-on time of the conventional IMRT planning was approximately 205 min. The time to create an MCO-plan was on average 43 ± 12 min.

**Conclusions:**

MCO planning enables novice treatment planners to create high quality IMRT plans for HNC patients. Plans were created with vastly reduced planning times, requiring less resources and a short learning curve.

## Introduction

For patients with head and neck cancer (HNC), intensity-modulated radiotherapy (IMRT) has been demonstrated to reduce radiation induced complications, as compared to conventional radiation delivery techniques [1,2]. IMRT allows for dose distributions with curative intended dose to tumor tissue, with an attempt to minimize dose to organs at risk (OARs) related to late toxicities, such as xerostomia and dysphagia [3,4].

The trade-offs between the target(s) and the relative large number of OARs in the head and neck area cause the conventional treatment planning procedure to be cumbersome. The creation of a conventional IMRT plan requires an iteration loop of changing patient specific trade-off objectives and dose re-computations, and is therefore subjective. Subtle changes in one of the optimization parameters potentially yield profound effects to the overall dose distribution quality. Furthermore, conventional IMRT increases the planning time and contains a relatively long learning curve [5-7]. The transition from IMRT to VMAT could further increase planning times due to longer calculation times in VMAT planning. The increasing demand of IMRT and VMAT plans, however, requires efficient departmental workflows.

Recently, multicriteria optimization (MCO) has become commercially available for IMRT [8]. With MCO, a library of Pareto optimal plans is generated automatically emphasizing different trade-off objectives. Each library plan is optimal in a way that one objective can only be improved by deteriorating on others. The final treatment plan can be selected by interactively navigating across the pre-computed Pareto plans from which a deliverable plan is created.

Previous studies have demonstrated that MCO results in treatment plans that are superior in terms of planning time and dose distributions as compared to conventional IMRT plans [9-14]. Craft *et al.* demonstrated that with MCO, high quality IMRT plans for glioblastomas and pancreatic cancers can be created more efficiently [9]. Using MCO for prostate cancers, significant reductions in rectal dose were demonstrated by McGarry *et al*. [13]. However this came, to some extent, at the expense of less conformal tumor dose distributions and higher dose to the bladder. Voet *et al*. showed that fully automatically generated IMRT plans for HNC were superior in terms of improved plan quality and reduced workload, and were in 97% of cases selected by physicians in favor of manually generated IMRT plans [10]. Their study used prioritized optimization, resulting in one treatment plan only, and not requiring manual Pareto surface navigation.

Last years, the demand for IMRT has grown exponentially: for multiple treatment sites as well as *in-silico* planning comparative studies. To efficiently use departmental resources, while striving for high plan quality, we tested the hypothesis that less experienced dosimetry planners create clinically acceptable IMRT plans with a commercially available MCO system as good as conventional IMRT plans created by experienced planners. Therefore, twenty HNC patients previously treated with conventional IMRT were included in this study. Plan quality was reported in terms of planning time, dosimetric and normal tissue complication probability (NTCP) comparisons, and blinded plan ratings, performed by two radiation-oncologists (RO) expert in HNC radiotherapy (H.B. and R.S.).

## Methods and materials

### Patients, prescriptions and delineation

The study cohort consisted of twenty patients, of which 11 males and 9 females (median age 58; range: 46–65), diagnosed with stage II-IV squamous cell carcinoma of the head and neck, which were successively selected from a database of HNC patients included in a prospective standard follow up program. Patients were included with tumors originating in the retromolar trigonum, base of tongue, tonsillar region, soft palate, nasopharynx, piriform sinus, supraglottic larynx, and glottis larynx. All patients were previously treated with curatively intended radiotherapy (conventionally planned IMRT) either alone or combined with concomitant chemotherapy or cetuximab.

For each patient, a simultaneous integrated boost technique was planned comprising a total dose of 70 Gy to the planning target volume (PTV_boost_, in 2 Gy per fraction, 5 fractions per week and 7 weeks) and 54.25 Gy to the prophylactic PTV (PTV_prophylactic_, in 1.55 Gy per fraction). Both PTVs were created with 5 mm margins to the clinical target volume to account for geometrical uncertainties in the treatment process.

For treatment planning optimization, the brain, spinal cord and parotid glands were contoured. Additionally, ring structures of 1 and 6 cm around PTV_prophylactic_ were constructed to ensure steep dose fall-off between the PTVs and surrounding OARs. For planning evaluation, the following OARs related to swallowing dysfunction were contoured (according to guidelines described elsewhere [15]): the supraglottic larynx, pharyngeal constrictor muscles (PCM)s, esophageal inlet muscle, and the cricopharyngeal muscle.

### Treatment planning

#### Conventional IMRT

The clinically delivered IMRT plans were created by multiple experienced planners (minimal 5 years experience) using the Pinnacle^3^ treatment planning system (TPS) (version 9.0, Philips Healthcare, Andover, MA). All plans consisted of seven equispaced beams and were prioritized optimized in two steps. First, each plan was optimized to ensure sufficient target coverage according to the dose level prescriptions, without exceeding the maximum dose to the spinal cord and brain, which were constrained to 50 Gy and 60 Gy, respectively. Second, the dose to the parotid glands was reduced without deteriorating on target coverage. The most relevant IMRT parameters set were: a dose grid resolution of 0.4 × 0.4 × 0.2 cm^3^; maximum number of segments 84; minimum four monitor units per segment; minimum segment area four cm^2^; optimization type DMPO (direct machine parameter optimization); and a final adaptive convolution dose computation. In DMPO the conversion step of a fluence-based plan to a segmented machine deliverable plan is integrated in the optimization. This improves IMRT planning by avoiding deterioration of the dose distribution of a sequential fluence optimization followed by conversion to segments. During optimization, however, a fast approximate dose calculation method is applied which requires a clinical (adaptive convolution) dose calculation after the optimization has finished.

#### MCO planning

Three novice planners with no experiences in IMRT planning and minimal instruction to IMRT created the MCO-plans using the RayStation TPS (research version 2.4.11, RaySearch Laboratories AB, Stockholm). The software was installed on a 64-bit Windows desktop computer with an Intel Xeon 2.4 GHz processor and 24GB DDR3 RAM. Per plan, the same structure definitions were used as for the conventional IMRT plans. A template with *n* tradeoff objectives and constraints was developed, based on the experience of the Pinnacle^3^ plans, to input the MCO. A library of 2*n* plans was created based on this template. During the first *n* plans, each objective was optimized individually and these were denoted as the anchor plans. The (n + 1)^th^ plan is the balanced plan in which all objectives were partially considered. The additional plans were the so-called auxiliary plans and constructed towards improving the Pareto surface as much as possible [8]. The final dose distribution was selected by navigation across the Pareto surface using slider bars on clinical objectives. A list with clinical criteria was used during navigation that included criteria for minimum and maximum dose in the targets and maximum dose to organs at risk like the spinal cord. A table with colored markers visually showed which criteria were fulfilled so that the majority of clinically unacceptable plans could be avoided. No ROs were involved in the final plan selection. Hence, a deliverable plan was created by direct aperture optimization, using similar IMRT parameters as in the conventional IMRT plans (a dose grid resolution of 0.3 × 0.3 × 0.3 cm^3^; maximum number of segments 84; minimum four monitor units per segment; minimum segment area four cm^2^) and a final collapsed cone dose computation.

### Plan evaluation

All Pinnacle^3^ plans were exported to the RayStation system. For each patient, the conventional IMRT and MCO-plans were independently evaluated and blind-rated by two experts RO in HNC (H.B. and R.S.). Plan rating was performed using an in-house-developed assessment form, including 5-point scales [poor (1) – excellent (5)] for the following items (Table 1):

**Table 1:** **Overview of the conventional IMRT and MCO-plan quality scores and the inter-rater reliability**

	**Technique**			
	**Conventional IMRT**	**MCO**		
**Item**	**Mean (SD)**	**Mean (SD)**	***p-*** **value**	**ICC agreement (95% CI)**
1: Dose hot spots in PTV_boost_	4.7 (0.5)	4.7 (0.5)	1.00	0.26 (−0.36 – 0.6)
2: Dose hotspots in PTV_prophylactic_	4.4 (0.9)	4.6 (0.5)	0.15	0.51 (0.28 – 0.79)
3: Dose cold spots in PTV_boost_	3.9 (0.9)	3.5 (0.9)	0.01	0.57 (0.22 – 0.77)
4: Dose cold spots in PTV_prophylactic_	4.0 (1.2)	3.9 (1.1)	0.45	0.70 (0.45 – 0.84)
5: Conformity of 95% isodose around PTV_boost_	3.8 (1.0)	3.5 (0.8)	0.11	0.19 (−0.45 – 0.56)
6: Conformity of 95% isodose around PTV_prophylactic_	4.0 (1.1)	3.7 (1.1)	0.07	0.61 (0.29 – 0.79)
7: Maximum dose to spinal cord	4.9 (0.5)	5.0 (0.2)	0.28	0.83 (0.70 – 0.91)
8: Parotid gland dose	4.4 (0.7)	4.3 (0.7)	0.62	0.67 (0.40 – 0.82)
9: Dose in unspecified tissue	4.0 (0.8)	3.7 (0.8)	0.07	0.28 (−0.31 – 0.61)
10: General plan quality	3.8 (1.2)	3.6 (1.1)	0.29	0.44 (−0.33 – 0.69)
Total	41.7 (6.4)	40.4 (5.4)	0.15	0.65 (0.57 – 0.71)

The plan quality scores range from 1–5 with 5 the highest score. *Abbreviations*: *IMRT* intensity-modulated radiotherapy, *MCO* multicriteria optimization, *PTV* planning target volume, *SD* standard deviation, *ICC* intra-class correlation coefficient, *CI* confidence interval. Level of statistically significant differences was set to *p* < 0.005; Bonferroni correction with α = 0.05/10 questions.

- PTV dose hotspots, cold spots and conformity of the 95% isodose line around both PTVs
- spinal cord maximum dose, parotid gland dose and dose in unspecified tissue
- general plan quality

Additionally, general plan remarks were reported by the ROs. After rating all conventional IMRT and MCO-plans independently the plans were compared side-by-side and the ROs’ preferred plan was determined for each patient.

More quantitatively, plans were compared by means of planning time, dose-volume parameters, conformity index (CI_V95%_), and NTCP for xerostomia [16] and physician-rated grade II-IV dysphagia [4]. The CI_V95%_ was defined as the ratio of the volume enclosed by the 95% isodose and the volume of the PTV that received at least 95% of the prescription dose.

### Statistical analysis

The inter-rater reliability was derived by the intra-class correlation coefficient using a two-way random consistency model (ICC [2,1], IBM SPSS Statistics version 22). Criteria to interpret the ICC were set to: moderate (ICC values from 0.40 to 0.59), substantial (0.60 to 0.79), and almost perfect (0.80 to 1.00). Statistically significant differences between evaluation parameters were assessed using Wilcoxon signed-rank test and considered statistically significant at *p* < 0.05. For multiple structures a Bonferroni correction of *p* < 0.003 (α = 0.05/15 structures) was applied. This study was granted exemption from our institution’s ethical review board

## Results

In total, the radiation-oncologists rated 800 items on plan quality. The overall plan quality score indicates no difference between conventional IMRT and MCO-plans (conventional IMRT: 3.8 ± 1.2 vs. MCO: 3.6 ± 1.1, *p* = 0.29). The inter-rater reliability and the mean of the individual rated items are listed in Table 1. Dose cold spots in the PTV_boost_ were slightly lower rated for the MCO-plans. However, all plans fulfilled the prescribed dose levels. Substantial to almost perfect agreement among the ROs was observed for the following ratings: dose cold spots in PTV_prophylactic_ (ICC = 0.70), dose conformity around PTV_prophylactic_ (ICC = 0.61), maximum spinal cord dose (ICC = 0.83), and parotid gland dose (ICC = 0.67). The majority of plan ratings (87%) were within one-point difference between the ROs. In 1% of the ratings there was less consensus (three-point difference). This was mainly caused by different scorings of two conflicting parameters: dose conformity around the PTV and parotid gland dose sparing. The inter-rater reliability for all plan ratings was 0.65 (95% CI: 0.57–0.71), indicating substantial agreement between the ROs.

The distribution of ratings between the conventional IMRT and MCO-plans is shown in Table 2. Perfect agreement was observed in 57% of all ratings. In 36% of the cases the difference was one scored point. In 3% of the rated items a three-point difference between the conventional IMRT and MCO-plan was observed. Furthermore, the ROs selected the preferred plan (conventional IMRT or MCO-plan) per patient as 60%: 40%, indicating a slight preference for conventional IMRT.

**Table 2:** **Cross table indicating the plan quality scores for all items of the conventional IMRT and MCO-plans**

**Score**		**MCO plans**					**Total**
		**1**	**2**	**3**	**4**	**5**	
Conventional IMRT plans	1	0 (0%)	2 (1%)	1 (0%)	0 (0%)	0 (0%)	**3 (1%)**
	2	2 (1%)	13 (3%)	12 (3%)	4 (1%)	3 (1%)	**34 (9%)**
	3	0 (0%)	6 (2%)	9 (2%)	18 (5%)	7 (2%)	**40 (10%)**
	4	0 (0%)	9 (2%)	28 (7%)	82 (21%)	18 (5%)	**137 (34%)**
	5	0 (0%)	7 (2%)	8 (2%)	46 (12%)	125 (31%)	**186 (47%)**
**Total**		**2 (1%)**	**37 (9%)**	**58 (15%)**	**150 (38%)**	**153 (38%)**	**400 (100%)**

The plan quality scores range from 1–5 with 5 the highest score.

**Figure 1:**
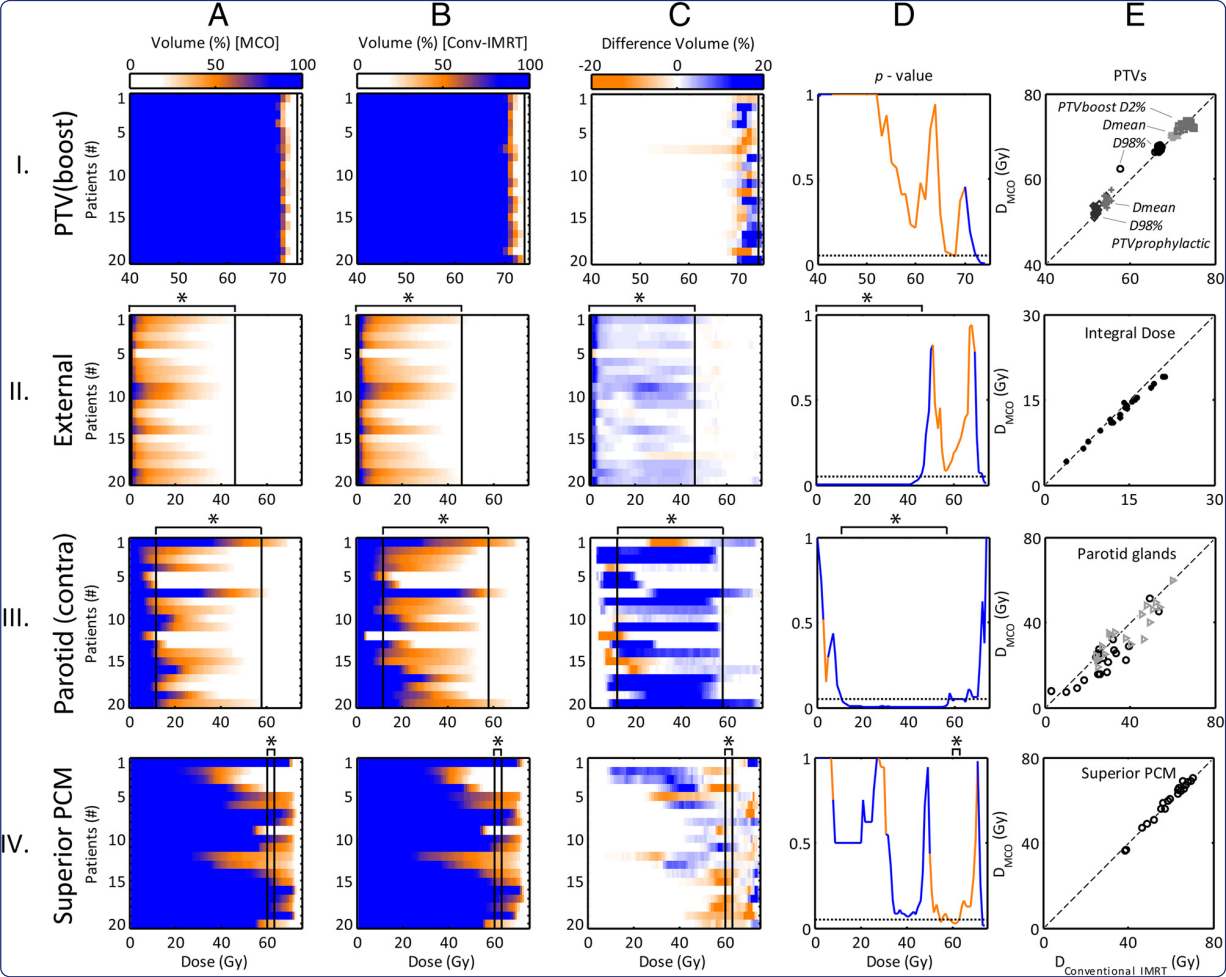
Colormap representation of cumulative DVHs of all patients (column **A**: MCO-plans and **B**: Conventional IMRT plans) and DVH difference maps (column **C**, conventional IMRT minus MCO) for the PTV_boost_ (row I), integral dose (i.e. External, row II), contra-lateral parotid gland (row III), and superior pharyngeal constrictor muscle (PCM, row IV). Each row in the colormaps indicates the relative volume (difference) against dose for a single patient. The vertical lines indicate the domain (*) at which the DVHs were significantly different. A comparison of DVHs for the conventional IMRT and MCO-plans resulted in a *p-*value (Wilcoxon signed-rank test) at each dose level (column **D**). The orange and blue line sections indicate a lower mean dose for the conventional IMRT or MCO-plans, respectively. The dotted line indicates the level of being statistically significant at *p* = 0.05. The mean dose per structure and per planning technique is plotted in column **E**. Additionally, the D_98%_ and D_2%_ of PTV_boost_ and D_98%_ of PTV_prophylactic_ is shown. The mean dose of the contra-lateral and ipsi-lateral parotid glands are illustrated by circles and triangles, respectively. The dashed line indicates the unity line.

Table 3 lists all dosimetric values, NTCP estimates, plan evaluation parameters and statistical results. For both the conventional IMRT and MCO-plans cumulative DVHs as well as DVH difference maps of the PTV_boost_, external (i.e. integral body dose), contra-lateral parotid gland and the superior pharyngeal constrictor muscle (PCM) are illustrated in Figure 1. A comparison of DVHs for the two types of plans resulted in a *p*-value at each dose level (Figure 1D). Furthermore, scatter plots show the mean dose values of the given structures, the D_98%_ of both PTVs and the D_2%_ of PTV_boost_, of the conventional IMRT and MCO-plans (Figure 1E).

**Table 3:** **Plan evaluation parameters and dose statistics**

**Parameters**	**Conventional IMRT**	**MCO IMRT**	**Difference**	***p*** **-value**
**Dose-volume values (Gy)**				
PTV (boost) D98%	66.4 ± 2.1	66.9 ± 1.1	−0.5 ± 0.5	0.03
PTV (boost)	70.2 ± 0.5	70.1 ± 0.3	0.2 ± 0.1	0.33
PTV (boost) D2%	73 ± 1.2	72.4 ± 0.7	0.6 ± 0.3	0.04
PTV (prophylactic) D98%	52 ± 0.7	52.5 ± 1.2	−0.4 ± 0.3	0.07
PTV (prophylactic)	54.6 ± 0.5	54.8 ± 1.0	−0.2 ± 0.2	0.53
Integral dose	**13.9 ± 4.5**	**12.9 ± 4.0**	**0.9 ± 1.4**	**<0.001**
Ring 1 cm around PTV	46.2 ± 2.6	45.0 ± 2.2	1.1 ± 0.8	0.008
Ring 6 cm around PTV	**16.9 ± 3.2**	**15.0 ± 2.5**	**1.9 ± 0.9**	**<0.001**
Spinal cord (D1%)	45.3 ± 3.9	44.1 ± 4.4	1.2 ± 1.3	0.17
Brain D1%	27.8 ± 14.5	27.5 ± 16.1	0.2 ± 4.8	0.31
Parotid gland (contra)	**28.1 ± 11.8**	**23.0 ± 11.2**	**5.1 ± 8.1**	**<0.002**
Parotid gland (ipsi)	39.1 ± 11.7	36.0 ± 11.1	3.0 ± 11.9	0.02
Superior PCM	58.8 ± 9.5	58.9 ± 10.2	−0.1 ± 3.1	0.91
Middle PCM	55.3 ± 14.0	56.3 ± 12.1	−1.0 ± 4.1	0.79
Inferior PCM	51.2 ± 16.8	52.2 ± 15.9	−1.0 ± 5.2	0.46
Supraglottic larynx	55.5 ± 15.3	57.3 ± 13.7	−1.9 ± 4.6	0.02
Esophagus inlet muscle	44.8 ± 12.2	44.1 ± 11.0	0.7 ± 3.7	0.28
Cricopharyngeus	48.6 ± 13.8	48.5 ± 13.6	0.1 ± 4.3	0.73
**NTCP values (%)**				
Xerostomia [contra]	**27.1 ± 20.9**	**18.7 ± 19.2**	**8.4 ± 7.4**	**<0.003**
Xerostomia [ipsi]	48.2 ± 25.8	41.8 ± 24.0	6.4 ± 14.3	0.03
Dysphagia	35.4 ± 14.7	36.9 ± 14.7	−1.5 ± 4.7	0.11
**Plan evaluation**				
CI 95% (PTV boost)	1.30 ± 0.12	1.28 ± 0.07	0.02 ± 0.03	0.98
CI 95% (PTV prophylactic)	1.54 ± 0.15	1.55 ± 0.13	−0.02 0.04	0.65
Monitor Units (#)	**590 ± 135**	**672 ± 157**	**−82 ± 46**	**0.02**
Total planning time (min)	205	43 ± 12	162	-

*Abbreviations*: *PTV* planning target volume, *PCM* pharyngeal constrictor muscle, *NTCP* normal tissue complication probability, *CI 95%* conformity index of 95% isodose with PTV. Items with statistically significant differences (structure related: *p <* 0.003 else: *p* < 0.05) in bold.

For the targets, MCO led to significantly lower volumes receiving >73Gy (Figure 1 row I). Furthermore, MCO-plans showed higher D_98%_ and lower D_2%_ values for PTV_boost_, indicating increased target dose uniformity (Table 3). For the external the V_<46Gy_ (i.e. the relative volume receiving a dose of 46 Gy or less) and the D_mean_ was significantly lower for the MCO-plans (Figure 1 row II and Table 3). Also, steeper dose fall-offs around the PTV_prophylactic_ was observed as indicated by the decreased mean dose to the 1 and 6 cm ring structures (Table 3). The CI95% for both PTVs showed no differences. The V_>11Gy_ and the D_mean_ of the contra-lateral parotid gland was significantly lower for the MCO-plans (Figure 1 row III and Table 3). For the superior PCM, the volume receiving approximately 60 Gy slightly increased for the MCO-plans (Figure 1 row IV).

**Figure 2:**
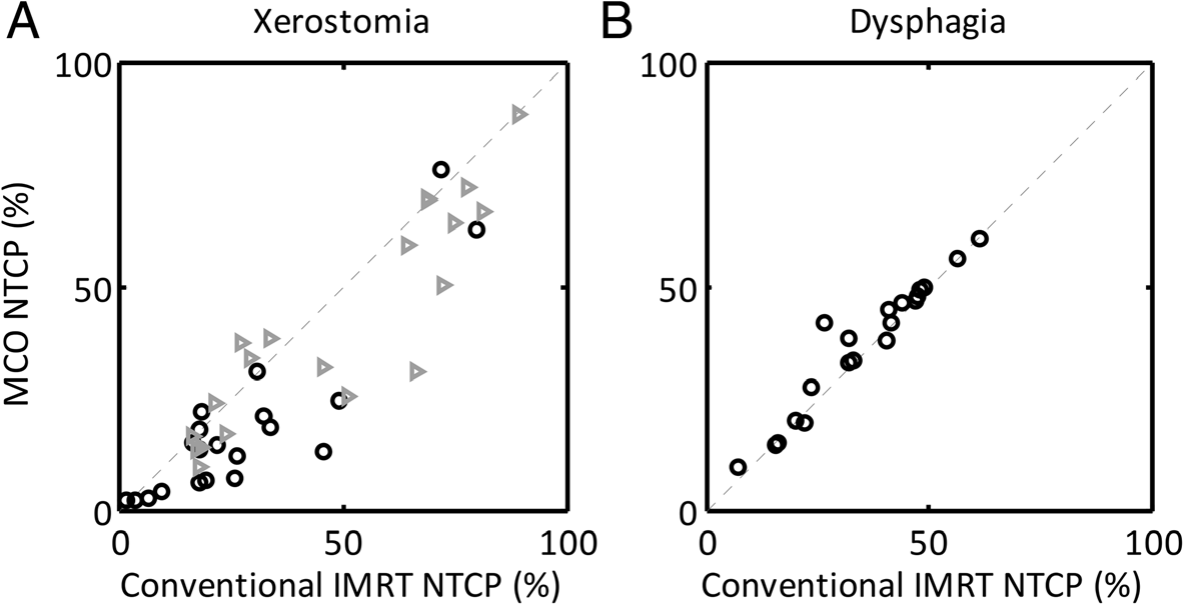
Normal tissue complication probability (NTCP) estimates for xerostomia (**A**) and physician-rated grade II-IV dysphagia (**B**). Xerostomia NTCP values were derived for the contra- (circles) and ipsi-lateral (triangles) parotid glands.

The number of monitor units increased using MCO planning (590 ± 135 vs. 672 ± 157, *p* = 0.02), indicating a higher degree of intensity modulation (Table 3). However, decreased integral body dose was observed which suggests that the MLC segments were generally smaller in the MCO-plans. For the presented cases, predictions of xerostomia were significantly lower (based on the mean dose to the contra-lateral parotid gland: 8.4 ± 7.4%, *p* < 0.003) for the MCO-plans (Figure 2A and Table 3). In contrast, NTCP-values for dysphagia were similar between both planning techniques (Figure 2B). However, this was expected, since no trade-off objectives were used on the structures related to swallowing dysfunction.

The hands-on time of the conventional IMRT planning was approximately 205 min, excluding time for contouring. The planning time included: beam configuration, planning parameter setup, generation of planning support structures (e.g. ring structures), and the trial-and-error inverse planning process. MCO planning took on average 43 ± 12 min. The active planning time was approximately 5 min for preparation and 20 min for navigating the Pareto surface.

## Discussion

The increased demand of IMRT for different treatment sites, as well as e.g. *in-silico* comparative planning studies, requires increased commitment of departmental resources. In this study, we demonstrated that an MCO system allows less experienced dosimetry planners to efficiently create high quality IMRT plans comparable to conventionally optimized IMRT plans for HNC.

The MCO-plans were created by three novice treatment planners. Prior to this study, these planners were introduced to IMRT planning, including a practical exercise on 5 MCO cases. In contrast, the conventionally optimized IMRT plans were created by multiple experienced IMRT planners, therefore not biasing the results as if the plans were created by one planner. Moreover, these plans were representative of the plan quality within the department. To assess the learning curve of MCO the quality of the MCO plans as planned by the novice planners would preferably be compared against MCO plans planned by experienced MCO planners. Moreover, the learning curve for MCO planning is relatively short, likely leading to a more constant plan quality.

The quantitative dosimetric comparisons revealed reduced dose to the parotid glands, steeper dose fall-off around the PTVs, and less integral body dose for the MCO-plans. However, these findings were not observed analyzing the ratings of the ROs (Table 1). This may be caused by the fact that all plans were blind-rated and that the ROs were not aware of any further possible dose reductions to critical regions. Furthermore, the dose to the swallowing structures (e.g. supraglottic larynx and pharyngeal constrictor muscles) slightly increased for the MCO-plans. However, these structures were not accounted for during planning optimization.

In 60% of cases the ROs selected the conventional IMRT plans over the MCO-plan. For some cases the selection was difficult because of the relatively small differences between the plans. Another reason for the RO of not selecting the MCO-plans was the underdosage of the PTV near the parotid glands for some plans. Slightly changing the slider bars on the parotid gland trade-off objectives would have resulted in different plans. Improving target coverage near the parotid glands would consequently deteriorate on parotid dose, and the presented NTCP-value reductions for xerostomia may be too optimistic for some cases. Therefore, instructions to the planner navigating the Pareto surface is crucial and could lead to plans which are more preferred by the RO. Moreover, the ROs may explore the Pareto surface, directly selecting the preferred plan for the individual patient. This benefit was discussed in more detail by Craft *et al*. [9].

In a number of cases, the MCO dose distribution quality decreased after the conversion step of the navigated fluence-based plan to a segmented machine deliverable plan. This conversion step resulted in decreased target coverage and increased dose to the OARs. To prevent the decreased plan quality, Fredriksson and Bokrantz proposed an approach to deliverable Pareto surface navigation, based on subsets of apertures identical across plans [17]. The proposed method is expected to lead to improved deliverable treatment plans (i.e. what you see is what you get). In conventional IMRT planning, on the other hand, decrease of plan quality can also occur when the clinical dose calculation method is applied after optimization.

It would be interesting to know if our findings are generally valid and could also be obtained with other MCO systems. The study of Craft *et al*. demonstrated vastly reduced planning times and improved dose distribution quality with MCO-plans for glioblastoma and pancreatic cancers [9]. The total time spend by the treatment planner and the RO was approximately 20 min. Prostate treatment planning time with MCO took approximately 60 min per case [11]. In our study, the mean planning time for HNC MCO planning was 43 min. The difference is mainly caused by the difference in time to create and navigate the Pareto plan library. Improvements in computer architecture, e.g. by calculation on the GPU, could reduce the time to create the Pareto plan library. In the study of Craft *et al*. [9] the Pareto surface was navigated by the physician, while in the study of Wala *et al*. [11] and in ours, no physician was involved in the selection of the final plan. The planning time with the MCO system was considerably lower than the time required to create a conventional IMRT plan (205 min). The latter was estimated based on the current clinical workflow and therefore representative for the included cases. Furthermore, this time was comparable to the manual planning time (198 min) reported by Voet *et al*. [10].

With interactive navigation across the Pareto surface the understanding of the dosimetric options available per patient will increase. This will hopefully lead to improved dose distributions with lower NTCP estimates, especially for HNC patients in which a relative high number of OARs are in close vicinity of the targets. Dose to these critical (sub)volumes may lead to a substantial variety of treatment-related adverse effects, such as xerostomia and dysphagia [1]. For example, it has been shown that long-term swallowing dysfunction after HNC radiotherapy is related to the mean dose of the larynx and pharyngeal musculature [4]. To reduce the corresponding NTCP estimates without deteriorating on target coverage, dose should be optimally balanced among the related OARs. Recently, it has been shown that direct application of multivariable NTCP models (including multiple dose parameters and prognostic clinical factors) in the optimization process facilitates individualized planning optimization in HNC [18]. To increase the MCO performance, a slider based on multivariable NTCP models would be preferred to find the optimal treatment plan. Moreover, this would reduce the number of currently used trade-off objectives to a few NTCP-based objectives, simplifying the navigation process.

In conclusion, we demonstrated that MCO-plans for HNC patients, created by novice dosimetry planners, result in IMRT plans with increased target dose uniformity and reduced dose to the parotid glands as compared to conventionally planned IMRT. ROs blind-rated the MCO-plans of similar quality as the conventional IMRT plans. Moreover, the MCO planning required less resources and a short learning curve. Therefore, MCO can serve as a promising tool to efficiently use the departmental resources.
